# A Flexible, Antibacterial Platform: Silver-Tuned Polyvinyl Alcohol with Enhanced Opto-Mechanical and Electrical Properties

**DOI:** 10.3390/polym18030415

**Published:** 2026-02-05

**Authors:** Abdelazim M. Mebed, Ali H. Mohsen, Diyar J. Hassan, Nadia A. Ali, Seenaa I. Hussein, Farah T. M. Noori, Alaa M. Abd-Elnaiem, Alhafez M. Alraih, Randa F. Abdelbaki

**Affiliations:** 1 Department of Physics, College of Science, Jouf University, P.O. Box 2014, Sakaka 72341, Saudi Arabia; 2 Department of Physics, College of Science, University of Baghdad, Baghdad 10071, Iraq; ali.muhsen1104@sc.uobaghdad.edu.iq (A.H.M.); nadia.ali@sc.uobaghdad.edu.iq (N.A.A.); seenaa.hussein@sc.uobaghdad.edu.iq (S.I.H.); farah.noori@sc.uobaghdad.edu.iq (F.T.M.N.); 3 Clinical Laboratory Sciences, College of Pharmacy, University of Baghdad, Baghdad 10071, Iraq; diyar.j@copharm.uobaghdad.edu.iq; 4 Physics Department, Faculty of Science, Assiut University, Assiut 71516, Egypt; 5 Department of Chemistry, College of Science, King Khalid University, Abha 61413, Saudi Arabia; alalshafee@kku.edu.sa; 6 Chemistry Department, Faculty of Science, New Valley University, El-Kharja 72511, Egypt; randafouad_1974@sci.nvu.edu.eg

**Keywords:** Ag/PVA nanocomposites, flexible electronics, optical band gap, mechanical strength, electrical conductivity, surface wettability, antibacterial activity, industrial diversification, communicable disease

## Abstract

Silver/polyvinyl alcohol (Ag/PVA) nanocomposite films were synthesized via solution casting with varying concentrations of Ag nanoparticles (1–5 wt%). A comprehensive investigation was conducted to understand the influence of Ag content on the structural, optical, mechanical, thermal, electrical, and antibacterial properties of the composites. UV-Vis spectroscopy revealed a red shift in absorption peaks and a reduction in the optical band gap, which decreased from 3.78 eV for pure PVA to 3.37 eV for the 5 wt% Ag composite. FTIR and SEM analyses confirmed successful nanoparticle incorporation and morphological changes. The nanocomposites exhibited enhanced tensile strength, elongation at break, Young’s modulus, and hardness due to strong interfacial interactions. The addition of Ag also increased hydrophobicity and imparted effective antibacterial activity. The electrical and thermal properties showed significant improvement: AC conductivity increased from 5.8 × 10^−9^ to 1.01 × 10^−4^ S/cm with Ag content, while the dielectric constant decreased. A high DC conductivity of 1.5 × 10^5^ S/cm was achieved with only 3 wt% Ag. Thermal conductivity also rose from 0.27 W/m·K for pure PVA to 0.92 W/m·K for the 5 wt% composite. These results demonstrate that Ag/PVA nanocomposites are promising multifunctional materials for flexible electronics, combining tunable optoelectronic properties with enhanced mechanical, thermal, and antibacterial performance.

## 1. Introduction

Polyvinyl alcohol (PVA) has garnered significant attention as a host polymer due to its excellent film-forming ability, high optical transparency, biocompatibility, and water solubility [1,2,3]. Its semi-crystalline nature, derived from hydroxyl groups and hydrogen bonding, allows it to be processed into various forms such as powders, films, and fibers, facilitating its use in diverse applications from industrial adhesives and emulsifiers to biomedical devices like drug delivery systems and artificial tissues [3,4].

Silver (Ag) nanoparticles (NPs) are equally prominent in modern technology due to their unique optical, electrical, and antimicrobial properties, which stem from their high surface-area-to-volume ratio and distinctive surface plasmon resonance (SPR) [5,6]. These characteristics make them valuable for a broad range of applications, including enhanced spectroscopic techniques like surface-enhanced Raman scattering, catalytic processes, and biomedical uses such as wound healing and anticancer treatments [6,7,8]. However, Ag NPs are prone to oxidation and aggregation, which can diminish their functionality. A polymer matrix like PVA is ideal for stabilizing the Ag NPs, preventing aggregation while also leveraging the reducing capability of its hydroxyl groups for synthesis [9,10].

The integration of Ag NPs into PVA creates a multifunctional nanocomposite with enhanced properties. The metal-polymer synergy can lead to improved thermal stability, mechanical strength, and electrical conductivity, making Ag/PVA systems a focus for advanced material applications [11]. Previous studies have demonstrated the feasibility of such composites. For instance, Faraji et al. [12] synthesized Ag/PVA nanocomposites via a rapid precipitation method, observing a red shift in the SPR peak with increasing reducing agent concentration. Similarly, Salloom et al. [3] prepared Ag/PVA films by solution casting, noting that higher AgNO_3_ concentrations led to a red shift in absorption and a reduction in the optical band gap. Also, several studies have extensively explored Ag/PVA nanocomposites, demonstrating their versatility through various synthesis methods and applications. For example, studies have utilized γ radiation [13], ternary composites with polyaniline [14], and green biological synthesis [15,16,17] to create materials for optoelectronics, advanced wound dressings, and water treatment. Furthermore, these composites have shown promise in functional devices like Schottky photodiodes [18] and triboelectric nanogenerators for energy harvesting [19,20], as well as in biomedical coatings [21]. While foundational work on chemically synthesized systems exists, it often reports modest electrical conductivity enhancements at low Ag concentrations [22,23] or explores post-synthesis modifications like heat treatment that can increase resistivity [24].

Beyond flexible electronics, polymer nanocomposites are critically important for antimicrobial and antiviral applications. Recent advances highlight how nano-fillers can impart bioactive functionality. For instance, nanocomposites incorporating metal–organic frameworks (MOFs) have demonstrated structure-dependent antibacterial activity, where the release of metal ions and the generation of reactive oxygen species are key mechanistic pathways [25]. Similarly, studies on quaternary ammonium-functionalized polymers reveal how tailoring the polymer’s chemical structure, specifically, the architecture and density of cationic groups, directly governs its biocidal efficiency by disrupting microbial membranes [26]. These studies underscore a fundamental principle: the bioactivity of a nanocomposite is not merely an additive property but arises from specific structure–property–bioactivity relationships. Although the Ag/PVA system is well-known for its antibacterial properties, a comprehensive study that correlates its physical property enhancements (optical, mechanical, electrical) with its biological activity, all as a function of Ag NPs concentration, remains less explored. Such a study could bridge the understanding between material design for functional electronics and bioactive applications.

Although Ag/PVA nanocomposites are widely studied, the literature frequently focuses on complex systems or specific applications. For instance, studies have used gamma radiation [13], incorporated ternary components such as polyaniline [20], or employed green biological synthesis [27] for applications ranging from wound dressings to Schottky diodes [13]. Other works involve post-synthesis modifications, such as heat treatment, which can alter electrical properties via Coulomb blockade [28]. In contrast, the present study deliberately employs a straightforward, room-temperature solution casting of a binary Ag/PVA system. This intentional simplicity is the key to its novelty: it allows for the isolation and systematic investigation of the fundamental structure–property relationships governed primarily by Ag NPs concentration (1–5 wt%). This approach is distinct from studies that modify properties through complex chemistry or post-processing; here, property tuning is achieved solely through systematic variation of a single, well-defined compositional variable. By avoiding ternary additives or complex post-processing, this work provides a clear and direct correlation between Ag NPs loading and the enhancement of optical, mechanical, thermal, electrical, surface, and antibacterial properties. The primary contribution is this comprehensive, multi-property dataset derived from a simplified model system. This dataset fills a significant gap in the literature by establishing a unified, foundational framework that maps how a single variable controls a suite of interconnected properties. This understanding is critical for the rational design of these nanocomposites, not only for flexible electronics but as a platform material for various advanced applications.

## 2. Materials and Methods

### 2.1. Chemicals

PVA with a molecular weight of 120,000–186,000 g/mol and AgNO_3_ (99.998%) were procured from Sigma-Aldrich, St. Louis, MO, USA. All materials were used as received without further purification.

### 2.2. Synthesis of Ag/PVA Nanocomposite

Pure PVA and 1–5 wt% Ag/PVA nanocomposite films were synthesized by solution casting. In this study, the Ag NPs concentration range of 1–5 wt.% was selected to systematically study the evolution of multifunctional properties. This range is frequently reported in the literature for binary Ag/PVA systems [29], and represents a critical window where significant enhancements in optical, mechanical, and electrical properties are achievable without inducing excessive aggregation or compromising the film’s processability and intrinsic flexibility. First, a homogeneous PVA solution was prepared by dissolving 2 g of PVA powder in 50 mL of deionized water under continuous stirring at 70 °C. This was followed by ultrasonication for 30 min using a Soniprep-150 MSE homogenizer to ensure complete dissolution and deaeration. Subsequently, different volumes of an AgNO_3_ aqueous solution were added to 10 mL aliquots of the PVA solution to achieve final Ag NP concentrations of 1, 3, and 5 wt%. In this process, the Ag NPs were formed in situ. The hydroxyl (–OH) groups of the PVA chains served a dual role: (*i*) as a reducing agent to convert Ag ions (from AgNO_3_) into metallic Ag^0^ NPs, and (*ii*) as a capping/stabilizing agent to prevent nanoparticle aggregation and oxidation [30]. A noticeable color change was observed, indicating the formation of Ag colloids stabilized within the PVA matrix. The mixture was stirred vigorously to ensure uniform dispersion of the Ag NPs. A visible color change confirmed the formation and stabilization of Ag colloids within the PVA polymer matrix. The resulting solutions were then cast into Petri dishes and allowed to dry at ambient temperature (~30 °C) for 72 h, yielding freestanding films with a thickness of 70–80 µm.

**Figure 1:**
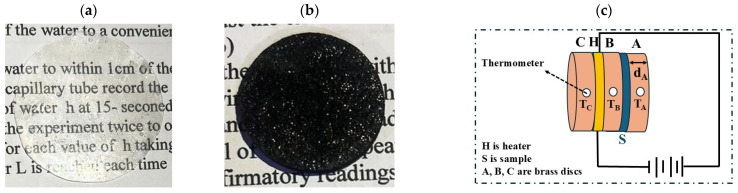
Photographic images of (**a**) pure PVA and (**b**) 5 wt% Ag/PVA composite films. (**c**) Schematic diagram of Lee’s disk apparatus for measuring thermal conductivity (reprinted from Ref. [31]).

Photographic images of the freestanding films were captured under ambient lighting using a digital camera to document their macroscopic appearance. The pure PVA film was transparent, whereas the Ag/PVA composite films were visually distinct; the 5 wt% composite exhibited a characteristic dark hue, as shown in Figure 1a, and Figure 1b, respectively.

### 2.3. Characterization Techniques

#### 2.3.1. Fourier Transform Infrared Spectroscopy

Chemical bonding and functional groups in the Ag/PVA composites were identified using Fourier transform infrared (FTIR) spectroscopy with a PerkinElmer Nicolet iS10 spectrometer (Waltham, MA, USA). Spectra were recorded over the range of 400–4000 cm^−1^ at a resolution of 2 cm^−1^.

#### 2.3.2. Scanning Electron Microscopy

The surface morphology of the Ag/PVA nanocomposites was examined using a field emission scanning electron microscope (FE-SEM) model FEI Inspect S50 scanning electron microscope (Thermo Fisher Scientific, Waltham, MA, USA) operating at an accelerating voltage of 25 kV. Before imaging, samples were sputter-coated with a thin gold layer to enhance surface conductivity.

#### 2.3.3. UV-Vis Spectroscopy

The optical properties of the films were analyzed with an SPUV-26 double-beam spectrophotometer (SCO Tech, Dingelstädt, Germany). Absorbance spectra were collected across the wavelength range of 300–800 nm.

#### 2.3.4. Mechanical Properties

Tensile strength ($T_{s}$), strain (S), and Young’s modulus ($Y_{M}$) were determined according to the ASTM D-882 standard [32] using a universal testing machine (Instron, Norwood, MA, USA) equipped with a 5 kg load cell. Rectangular film specimens (10 mm × 80 mm) were tested at a crosshead speed of 10 mm/min. The numerical value of $Y_{M}$ for Ag/PVA was calculated using Equation (1):

(1) YM=TsS=FA∆l∆l0

where *F* is the applied force, *A* is the cross-sectional area, $l_{0}$ is the initial gauge length, and ∆l is the elongation. The hardness of the composite films was measured using a Shore A durometer (PTC Instruments, Los Angeles, CA, USA) in accordance with ASTM D2240 [33].

#### 2.3.5. Dielectric and Electrical Properties

Dielectric properties, including the dielectric constant (ε′), dielectric loss (ε″), and AC conductivity ($\sigma_{ac}$), were measured using an Agilent E4980A precision LCR meter (Santa Clara, CA, USA) over a frequency range of 10^2^ to 10^6^ Hz at room temperature. The dielectric constant ($\varepsilon^{\prime }$) was calculated from the measured capacitance (*C*) using Equation (2) [34,35]:

(2) $$\varepsilon^{\prime }=\frac{Cd}{\varepsilon_{0}A}$$

where d is the sample thickness, *A* is the electrode area, and $\varepsilon_{0}$ is the vacuum permittivity. The AC conductivity, $\sigma_{ac}$, was derived from the dielectric loss using:

(3) $$\sigma_{ac}=2\pi f\varepsilon_{0}\varepsilon^{\prime \prime }$$

The complex electric modulus (M), defined as $M\;=\;M^{\prime }\;+\;iM^{\prime \prime }$, was also calculated to analyze interfacial polarization, with the real and imaginary parts given by:

(4) $$\left.\begin{array}{c} M^{\prime }=\frac{\varepsilon^{\prime }}{\varepsilon^{\prime 2}+\varepsilon^{\prime \prime 2}}\\ M^{\prime \prime }=\frac{\varepsilon^{\prime \prime }}{\varepsilon^{\prime 2}+\varepsilon^{\prime \prime 2}} \end{array}\right\}$$

#### 2.3.6. Thermal Conductivity

Thermal conductivity (K) was determined using Lee’s disc apparatus, shown schematically in Figure 1c [31,36]. The temperatures of brass discs A, B, and C ($T_{A}$, $T_{B}$, and $T_{C}$) were measured with a digital thermometer upon reaching thermal equilibrium. The heater was operated at 6 V and 0.25 A. The thermal energy transferred per unit area per second (ε) through a disk-shaped specimen, from which the thermal conductivity was determined using the following relationship [36,37]:

(5) $$K=\frac{\varepsilon [T_{A}+\frac{2}{r}(d_{A}+\frac{d_{s}}{4})T_{A}+\frac{d_{s}T_{B}}{2r}]}{\left(\frac{T_{B}-T_{c}}{d_{s}}\right)}$$

In this expression (Equation (5)), the parameter ε denotes the thermal energy flux, quantified in watts per square meter, and is defined by the following equation:

(6) $$\varepsilon =\frac{IV}{\pi r^{2}(T_{A}-T_{B})+2\pi r[d_{A}T_{A}+\frac{d_{s}(T_{A}-T_{B})}{2}{+d}_{B}T_{B}+d_{c}T_{c}]}$$

The variables are defined as follows: r represents the disk radius; $d_{A}$, $d_{B}$, and $d_{c}$ correspond to the thicknesses of the individual brass disks A, B, and C, respectively; and $d_{s}$ indicates the thickness of the sample under test.

#### 2.3.7. Surface Wettability

Surface wettability was evaluated by measuring the water contact angle (WCA) via the sessile drop method, following ASTM D-5946 [38]. A 10 µL droplet of deionized water was dispensed onto the PVA and Ag/PVA film surface using a micromanipulator-controlled syringe, and the static contact angle was measured. For each sample, WCA values were recorded for both the left and right sides of the droplet at multiple locations across the surface, and the average of these measurements is reported.

#### 2.3.8. Antibacterial Activity

The antibacterial activity of the Ag/PVA films was evaluated against *Escherichia coli* (Gram-negative) using the standard disc diffusion assay. Film samples were cut into 6 mm diameter discs, placed on Mueller-Hinton agar plates inoculated with the bacteria, and incubated at 37 °C for 6 h. The diameter of the inhibition zone around each disc was measured in millimeters.

## 3. Results and Discussion

### 3.1. FTIR Analysis

**Figure 2:**
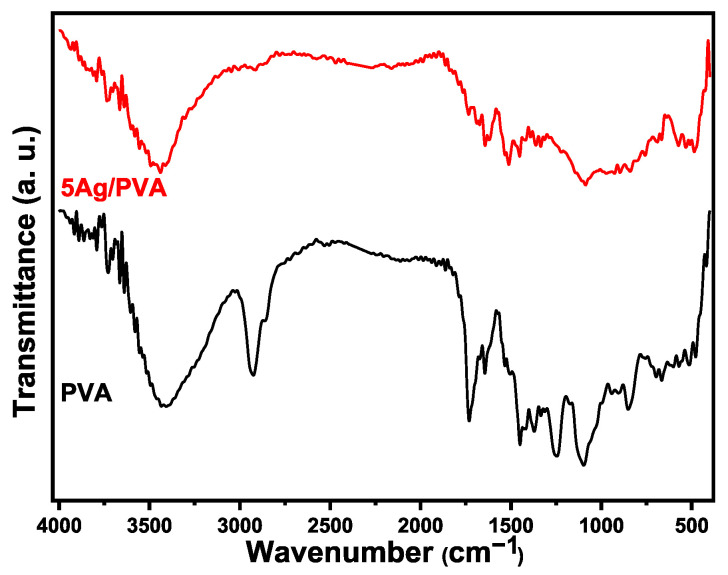
FTIR spectra of pure PVA and 5 wt% Ag/PVA nanocomposite.

The FTIR spectra of pure PVA and the 5 wt% Ag/PVA nanocomposite recorded at room temperature are presented in Figure 2. A comparative analysis of FTIR spectra reveals the fundamental vibrational modes of the PVA polymer and elucidates the molecular-level interactions introduced by the Ag NPs. The most prominent feature in the pure PVA spectrum is the broad absorption band centered around 3300 cm^−1^, which is characteristic of O–H stretching vibrations [39]. The breadth of this band signifies extensive intermolecular and intramolecular hydrogen bonding between the hydroxyl groups of adjacent polymer chains. Upon the incorporation of 5 wt% Ag NPs, subtle changes in this region suggest that the Ag NPs interact with these hydroxyl groups, potentially modifying the hydrogen-bonding network within the polymer matrix. The detected shift of the O–H stretching band (~3300 cm^−1^) for 5 wt% Ag/PVA composites indicates a modification of the hydrogen-bonding network within PVA due to interaction with Ag NPs.

The vibrational signatures of the polymer’s carbon backbone are clearly identified. The band observed at approximately 2930 cm^−1^ is attributed to the asymmetric stretching vibration of methylene (CH_2_) groups. Furthermore, the presence of carbonyl (C=O) and carbon-carbon double bond (C=C) functionalities is confirmed by the distinct peaks at 1710 cm^−1^ and 1652 cm^−1^, respectively [40]. These unsaturated bonds are of particular interest as they can act as active sites for the formation of polarons and bipolarons, which are crucial for charge transport in polymeric systems. The fingerprint region provides further evidence of successful nanocomposite formation. A significant spectral modification is observed in the Ag/PVA nanocomposite around the band at 1326 cm^−1^, which is associated with C–H wagging vibrations. For Ag/PVA composites, a noticeable decrease in the intensity ratio between this band and the one at 1429 cm^−1^ (assigned to CH_2_ bending) is evident. The detected decrease indicates a decoupling of the corresponding vibrational modes, most likely caused by coordination between the electron-rich oxygen of the PVA’s hydroxyl groups and the surface of the Ag NPs. The FTIR analysis is consistent with previously reported studies on metal-polymer composites [41]. Additional characteristic peaks further confirm the PVA structure: the C–O stretching vibration of carbonyl groups appears at 1105 cm^−1^, while the out-of-plane bending of C–H bonds is observed at 962 cm^−1^. The C–C stretching vibrations of the polymer backbone are visible at 845 cm^−1^. At lower wavenumbers, a broad band near 663 cm^−1^ corresponds to the wagging vibration of OH groups, and the weak features between 426 cm^−1^ and 486 cm^−1^ are related to C–O group deformations. Notably, the appearance of a distinct signal in the 370–845 cm^−1^ range in the nanocomposite spectrum can be attributed to the coordination bond formed between the silver atoms and the oxygen atoms of the PVA matrix, providing direct evidence of the successful synthesis of the Ag/PVA nanocomposite. Table 1 provides a comprehensive summary of the identified vibrational bands and their assignments for both pure PVA and the 5 wt% Ag/PVA nanocomposite.

The observed spectral modifications provide direct evidence for the nature of the interactions between the PVA matrix and the Ag NPs. The primary interaction is a coordination bond formed between the electron-donating oxygen atoms of the PVA hydroxyl (–OH) groups and the surface of the Ag NPs [29]. This is supported by the shift of the broad O–H stretching band, indicating a change in the hydrogen-bonding network of PVA as its hydroxyl groups engage with the nanoparticle surfaces. Furthermore, the notable change in the intensity ratio of the bands at 1429 and 1326 cm^−1^ suggests a decoupling of C–H and CH_2_ vibrational modes, which is consistent with the introduction of strong Van der Waals forces and physical adsorption at the polymer-nanoparticle interface. These combined interactions, coordination bonding, and Van der Waals forces are crucial for forming a coherent interface. They facilitate effective stress transfer for mechanical reinforcement, modify the local dielectric environment for optical property tuning, and create the interconnected network that underpins the enhancement of electrical and thermal properties in the nanocomposites.

### 3.2. Surface Morphology

**Figure 3:**
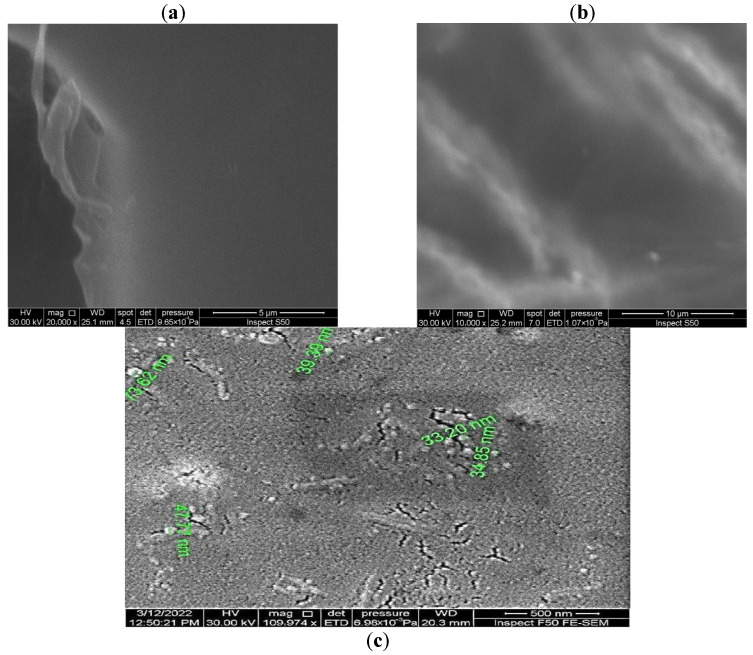
SEM micrographs of (**a**) pure PVA and (**b**,**c**) 5 wt% Ag/PVA nanocomposite film at different magnifications.

The surface morphology of the synthesized films was investigated using FE-SEM to assess the structural integration of Ag NPs within the PVA matrix. As a baseline, the SEM micrograph of pure PVA (Figure 3a) is consistent with our previous work and other studies [42]) reveals a characteristically smooth and homogeneous surface, which is a typical outcome of the solution casting method. In contrast, the FE-SEM image of the PVA nanocomposite loaded with 5 wt% Ag NPs, presented in Figure 3b,c, exhibits a distinct and transformed morphology. The surface displays a planar yet modified texture, punctuated by the presence of some nano-cracks. These cracks are not uncommon in polymer nanocomposites and can arise from internal stresses generated during the solvent evaporation and film-solidification process, potentially exacerbated by the incorporation of rigid inorganic fillers. A critical observation is the distribution and size of the Ag NPs within the polymer. The micrograph confirms a generally uniform dispersion of the Ag NPs, with minimal evidence of large-scale aggregation. This homogeneous distribution is a key indicator of the effectiveness of the synthesis protocol, particularly the ultrasonication step, in achieving a stable colloidal mixture prior to casting. Particle size analysis, performed on multiple visible nanoparticles in the image, reveals a size distribution ranging from approximately 13.62 nm to 39.39 nm, with a calculated average particle size of 33 ± 13 nm. The observed nanoscale dimension and the successful integration of the Ag NPs filler without severe phase separation are promising for the development of a continuous, multifunctional nanocomposite film, as they suggest a significant interfacial area for polymer-nanoparticle interactions, which is crucial for property enhancement.

### 3.3. Optical Properties

**Figure 4:**
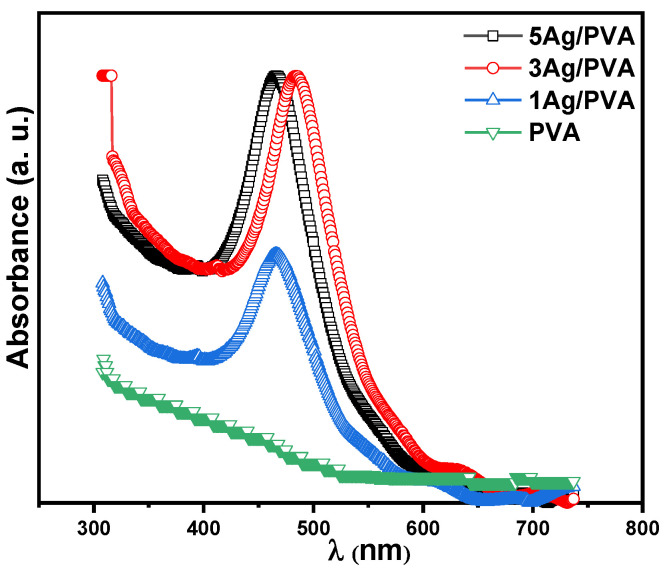
Absorbance spectra of pure PVA and Ag/PVA nanocomposites with different Ag NP concentrations, showing the evolution of the SPR peak.

The optical absorption characteristics of the pure PVA and Ag/PVA nanocomposite films were systematically investigated to understand their potential for optoelectronic applications. The absorbance spectra for the pure PVA and Ag/PVA nanocomposite films with varying Ag concentrations are presented in Figure 4. The absorbance spectrum of pure PVA revealed a high transparency and negligible absorption across the visible light spectrum (approximately 400–800 nm), which is consistent with its intrinsic wide band gap and colorless nature. This establishes a clear baseline against which the effect of Ag NPs can be evaluated. A dramatic alteration in optical behavior is observed upon the incorporation of Ag NPs. All Ag/PVA nanocomposite films exhibit a significant enhancement in absorbance, with the intensity scaling directly with the increasing weight percentage of Ag. This confirms that a higher precursor concentration successfully leads to a greater population of light-absorbing Ag NPs within the PVA matrix. The most definitive evidence for the formation of metallic Ag NPs is the emergence of a distinct absorption band in the range of 420–460 nm. This feature is absent in the pure PVA spectrum and is a signature of the localized surface plasmon resonance (LSPR) effect [22,43]. The LSPR occurs when the frequency of incident photons matches the collective oscillation frequency of the conduction electrons at the nanoparticle surfaces. Crucially, the LSPR peak not only intensifies but also undergoes a noticeable red shift (i.e., towards longer wavelengths) as the Ag content increases from 1 wt% to 5 wt%. The spectral shift can be attributed to several factors, including an increase in the average particle size of the filler, a decrease in inter-particle distances leading to stronger plasmonic coupling, and changes in the local dielectric constant of the polymer environment surrounding the nanoparticles. The observed behavior aligns well with established Mie theory for small metallic particles and underscores the successful tuning of the nanocomposite’s optical properties through compositional control [44].

The symmetric profile and narrow full width at half maximum (FWHM) of the surface plasmon resonance (SPR) band suggest the successful synthesis of nearly spherical Ag NPs [45]. The average particle diameter (d) was estimated from the FWHM ($∆E_{1/2}$) of the SPR band, based on a model that assumes free-electron behavior for the conduction electrons [46]. The calculation employed the following relation [47]:

(7) $$d=\frac{2v_{f}\hbar c}{∆E_{1/2}E_{SPR}^{2}}$$

where $v_{f}$ is the Fermi velocity of electrons in Ag (1.39 × 10^6^ m/s), ℏ is the reduced Planck’s constant, c is the speed of light, $∆E_{1/2}$ is the FWHM in energy units, and *E*_*SPR*_ is the energy at the SPR peak. The average diameter of the Ag NPs was calculated to be approximately 20 nm using this method. This value, which falls within a realistic nanoscale range, is consistent with the morphological observations from FE-SEM analysis.

The optical absorption coefficient (α) was calculated from the measured absorbance (A) and sample thickness (t) using the Lambert-Beer law [48]:

(8) $$\alpha =\frac{2.303A}{t}$$

In addition, the fundamental optical band gap ($E_{g}$), which is a critical parameter for optoelectronic applications, was determined using Tauc’s method [49]. This approach relates the absorption coefficient to the photon energy (hν) through the following equation:

(9) $$\alpha h\upsilon =B{\left(h\upsilon -E_{g}\right)}^{n}$$

where *B* is a constant related to the transition probability, and the exponent n defines the nature of the electronic transition (e.g., $n=\frac{1}{2}$ for direct allowed transitions) [50].

**Figure 5:**
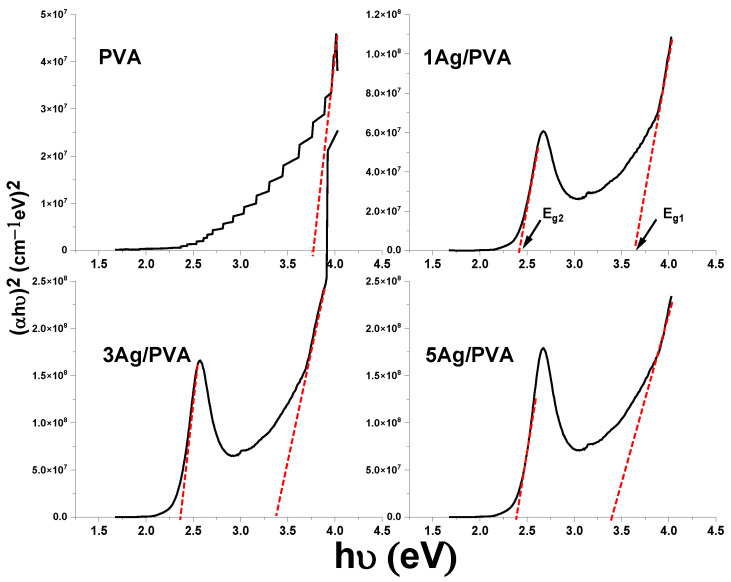
Tauc plots of (αhν)^2^ versus photon energy (hν) for PVA and Ag/PVA nanocomposites. The dashed line represents the linear extrapolation of the Tauc plot, from which the optical band gap energy is determined.

The Tauc plots, displaying (αhν)^2^ as a function of photon energy (hν) for pure PVA and Ag/PVA, are presented in Figure 5. The analysis reveals a significant modification of the PVA’s electronic structure upon the incorporation of Ag NPs. A distinct absorption feature in the UV region is associated with the intrinsic band gap of pure PVA. Notably, the absorption edge exhibits a gradual red shift with increasing Ag concentration, visually indicating a reduction in the optical band gap. This phenomenon is attributed to the interaction between the Ag NPs and the PVA chains, which facilitates the formation of localized electronic states within the band structure, thereby enhancing low-energy electronic transitions. A single optical band gap is observed for pure PVA, whereas the Ag/PVA composites exhibit two distinct band gaps. The appearance of two distinct optical band gaps (E_g1_ and E_g2_) in the nanocomposites suggests the presence of multiple electronic transition pathways. The lower-energy gap (E_g1_), which decreases from 2.41 eV to 2.37 eV with increasing Ag content, is assigned to the intrinsic band gap of the PVA matrix. Its systematic reduction is attributed to the introduction of defect states and a modification of the polymer’s electronic structure due to strong interaction with the Ag NPs [51]. The higher-energy gap (E_g2_), observed only in the nanocomposites, is likely associated with direct optical transitions originating from the Ag NPs. This could arise from interband transitions of Ag (e.g., from the d-bands to the conduction band near the X and L points of the Brillouin zone) or represent a higher-energy transition related to the LSPR of the Ag NPs, which can exhibit multiple resonance modes depending on size, shape, and local dielectric environment [52]. The concurrent presence and tuning of both gaps highlight the hybrid electronic nature of the Ag/PVA nanocomposite.

The calculated optical band gap values ($E_{g1}$ and $E_{g2}$), summarized in Table 2, provide quantitative confirmation of this trend. The band gap of pure PVA is determined to be 3.78 eV. The value of $E_{g1}$ is systematically decreased, reaching 3.37 eV for the 5 wt% Ag/PVA composite. Furthermore, the Ag/PVA nanocomposites exhibit a second, smaller energy gap ($E_{g2}$), suggesting the emergence of multiple electronic transition pathways. Two interconnected mechanisms can explain the observed reduction in the primary band gap. First, the incorporation of Ag introduces defect states within the HOMO-LUMO gap of PVA; the increasing density of these localized states with higher Ag content effectively narrows the energy gap required for electronic excitation [4,53]. Second, the increased structural disorder within the nanocomposite films contributes to the band gap narrowing, a behavior consistent with prior studies [4]. In contrast to the dual optical band gaps and direct transitions observed in our work, a previous study on chemically reduced Ag/PVA nanocomposites reported a single, indirect band gap, which decreased from 5.31 eV to 4.56 eV as the Ag content increased from 0 to 1.5 wt% [54]. A separate study utilizing laser ablation to synthesize Ag/PVA nanocomposites reported indirect optical band gaps of 4.87, 4.84, and 4.78 eV for different nanoparticle loadings [55]. This contrasts with our findings, where the composites exhibited a direct transition mechanism and significantly lower band gap values, decreasing to 2.37 eV, highlighting the profound influence of the synthesis method on the resulting optical properties. In agreement with the observation that Ag/PVA composites can exhibit dual optical band gaps, a study using a plasma-solution method reported both direct and indirect transitions, with values decreasing to 3.04 eV and 2.33 eV, respectively, after 20 min of processing [56]. This contrasts sharply with our results, where a simpler synthesis method produced a significantly lower direct band gap of 2.37 eV at a 5 wt% Ag loading. The considerable difference in the obtained band gap values underscores the critical role of the synthesis technique in tailoring the electronic structure of the nanocomposite.

The tunability of the optical band gap is directly linked to the SPR of the Ag NPs. SPR is typically observed between 400 and 800 nm, not only enhancing light–matter interactions but also playing a pivotal role in modifying the dielectric environment and electronic structure of the host polymer. The ability to precisely control the band gap by varying the Ag concentration underscores the potential of these nanocomposites for designing advanced optoelectronic devices, such as tunable photodetectors and SPR-based biosensors [57].

### 3.4. Mechanical Properties

**Figure 6:**
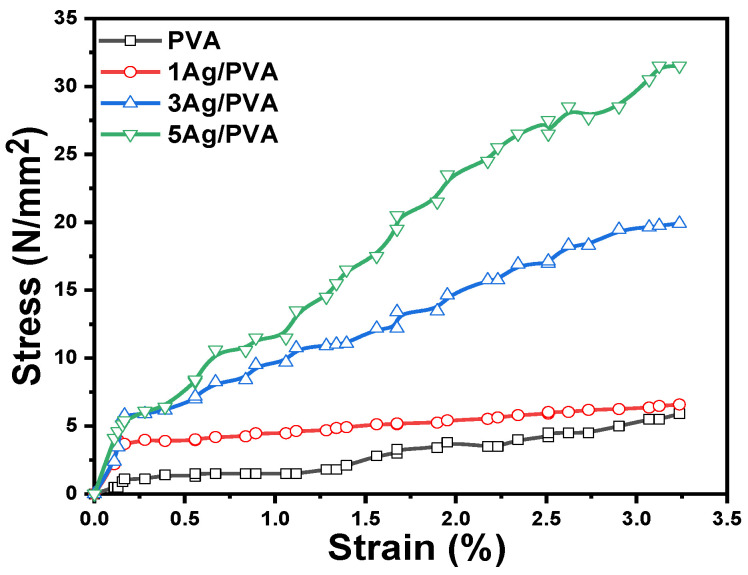
Stress–strain curves for pure PVA and Ag/PVA nanocomposites, illustrating the change in mechanical behavior with Ag content.

The mechanical integrity of the synthesized PVA and Ag/PVA films, all prepared with a consistent thickness of 70–80 μm, was rigorously evaluated to understand the reinforcing effect of Ag NPs on the PVA matrix. The stress–strain curves, presented in Figure 6, reveal a significant transformation in mechanical behavior induced by the Ag filler. Pure PVA film exhibited characteristic properties of a flexible but relatively weak polymer, with a tensile strength ($T_{s}$) of 4.1 N/mm^2^ and an elongation at break of 3.1%. The incorporation of Ag NPs dramatically altered the stress–strain profile. The addition of a small amount (1 wt%) of Ag led to a concurrent improvement in both strength (5.58 N/mm^2^) and ductility (3.3% strain), suggesting an initial enhancement in toughness. However, at higher loadings (3 and 5 wt%), a distinct trade-off emerged: while tensile strength surged remarkably to 19.92 N/mm^2^ and 30.5 N/mm^2^, respectively, the strain at break decreased. This indicates a transition in the material’s failure mode from ductile to more brittle, a classic signature of polymer reinforcement with rigid fillers. The stress–strain profiles for all compositions consistently display two distinct deformation regions. The initial linear segment, representative of elastic deformation, shows a progressively steeper slope with increasing Ag content. This directly correlates with a significant rise in Young’s Modulus ($Y_{M}$), which increased from 1.2 MPa for pure PVA to 2.4 MPa for the 5 wt% Ag/PVA composite, as detailed in the accompanying Table 2. The subsequent plastic deformation region becomes less pronounced in the high-content Ag composites, consistent with their increased brittleness. The comprehensive enhancement in mechanical strength and stiffness is attributed to the role of Ag NPs as multifunctional crosslinking agents. The Ag NPs form coordinated interactions and strong Van der Waals forces with the hydroxyl groups of the PVA chains, creating a percolating network within the matrix [58]. The network efficiently transfers applied stress and restricts polymer chain mobility, leading to higher modulus and strength, albeit at the cost of reduced ductility. The crosslinking mechanism is further corroborated by the Shore A hardness measurements, which showed a substantial increase from 52 for pure PVA to 68 for the 5 wt% Ag/PVA composite. The high surface-area-to-volume ratio of the well-dispersed Ag NPs, as confirmed by FE-SEM, maximizes their interfacial interaction with the polymer, which is the fundamental cause for the observed reinforcement of the nanocomposite’s mechanical properties [59].

A comparison with a previous study [54] highlights distinct mechanical behaviors between the two Ag/PVA nanocomposite systems. The referenced work, utilizing Ag loadings up to 1.5 wt%, reported a dramatic increase in $Y_{M}$ from 0.26 GPa for pure PVA to 4.8 GPa for the highest Ag content, an enhancement of over 1700%. This was accompanied by a significant increase in $T_{s}$ but a substantial reduction in strain at break, indicating a pronounced trade-off where high stiffness was achieved at the cost of ductility. In contrast, the nanocomposites synthesized in our present study demonstrate a more balanced enhancement. While our system also showed a consistent increase in $Y_{M}$ and $T_{s}$ with Ag content up to 5 wt%, the improvement was more moderate. More notably, our composites maintained a significantly higher strain at break, even at the highest Ag loading. The key difference suggests that our synthesis method, potentially through better nanoparticle dispersion or softer interfacial interactions, successfully reinforced the PVA matrix without inducing the same level of brittleness. The balance between improved strength and retained flexibility is a crucial advantage for applications in flexible electronics.

### 3.5. Thermal Conductivity

**Figure 7:**
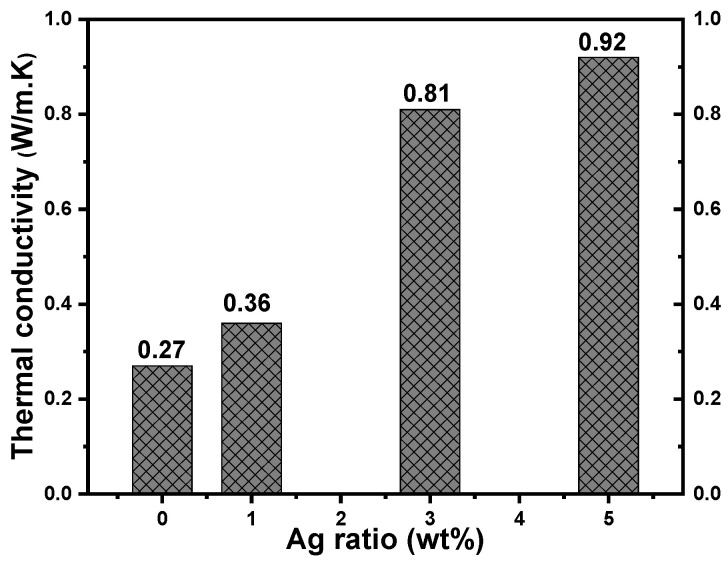
Thermal conductivity (K) as a function of Ag NPs concentration for Ag/PVA nanocomposites.

The thermal transport properties of the Ag/PVA nanocomposites were quantitatively assessed using Lee’s Disc method to determine the effect of Ag NPs inclusion. The results, illustrated in Figure 7, demonstrate a substantial improvement in thermal conductivity (K) with increasing Ag content. The base PVA matrix, an electrical and thermal insulator, exhibited a low intrinsic thermal conductivity of 0.27 W/m·K. The K value increased progressively to 0.36, 0.81, and 0.92 W/m·K for Ag loadings of 1, 3, and 5 wt%, respectively. This represents an overall improvement of over 240% for the 5 wt% composite compared to pure PVA. The dramatic enhancement is fundamentally attributed to the superior intrinsic thermal conductivity of Ag, which provides highly efficient pathways for phonon-mediated heat transport. The incorporation of Ag NPs introduces a multitude of additional heat carriers into the otherwise thermally resistant PVA polymer matrix. The most significant improvement observed at the highest loading (5 wt%) is likely due to the formation of a more interconnected and continuous thermally conductive network from Ag throughout the PVA. As the Ag NPs concentration increases, the inter-particle distance decreases, facilitating the creation of these percolating pathways that allow for more efficient heat flow than the mechanism of isolated particles within an insulating matrix.

The increase in K can be described as the establishment of metallic thermal bridges of Ag within the PVA polymer. The uniform distribution of Ag NPs, as suggested by prior analysis, is crucial for developing these effective heat transfer channels without significant phonon scattering at interfaces. The resulting composite transitions from being a thermal insulator to a material with moderately improved thermal management capabilities. The finding, which aligns with established composite theory, indicates that the Ag/PVA nanocomposites possess superior thermal performance, a critical characteristic for applications in flexible electronics where heat dissipation is a concern [60].

### 3.6. Electrical and Dielectric Properties

**Figure 8:**
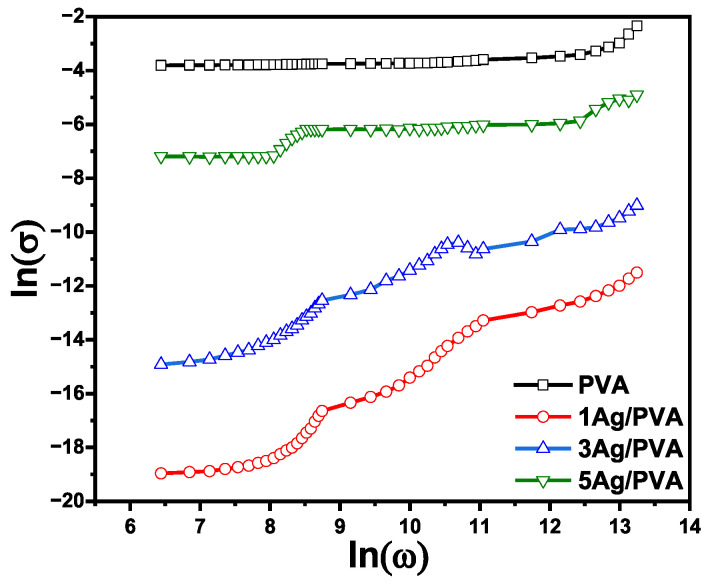
The logarithmic dependence of electrical conductivity (σ) on angular frequency (ω) for pure PVA and Ag/PVA nanocomposites with varying Ag content.

The electrical conductivity (σ) of the Ag/PVA nanocomposite films was systematically investigated, revealing a profound dependence on the concentration of Ag NPs. As summarized in Table 2 and illustrated in Figure 8, the incorporation of Ag NPs induces a dramatic enhancement in the electrical properties of the inherently insulating PVA matrix. The electrical conductivity increased by four orders of magnitude, from a baseline of 5.8 × 10^−9^ S/cm for the pure PVA to 1.01 × 10^−4^ S/cm for the 5 wt% Ag/PVA composite. The exponential rise is a classic signature of percolation behavior in conductor-insulator composites. At lower Ag concentrations (1–3 wt%), the Ag NPs are largely isolated within the PVA matrix. In this regime, electrical conduction is primarily governed by the quantum mechanical tunneling effect, where electrons “hop” through the insulating polymer barrier between separated Ag NPs. The probability of this tunneling is highly sensitive to the inter-particle distance. The improvement in electrical conductivity, a key consequence of embedding Ag NPs within the PVA matrix, is likely due to the Coulomb blockade mechanism [61].

As the Ag loading increases to 5 wt%, the average distance between nanoparticles decreases sufficiently to form interconnected, continuous conductive pathways or channels throughout the PVA polymer. The established a percolating network, allowing for direct charge transport and a massive leap in conductivity. The analysis of alternating current conductivity ($\sigma_{ac}$) provides further insight into the charge transport mechanisms. The observed frequency-dependent behavior, as seen in Figure 8, can be attributed to two main factors: the mobility of the polymer chains and the motion of ionic charge carriers. The rise in conductivity at higher frequencies is consistent with the response of free electrons within the newly formed metallic networks, while contributions at lower frequencies are often associated with interfacial polarization effects at the boundaries between the conductive Ag NPs and the insulating PVA matrix. The overall enhancement is thus attributed to a synergistic effect of increased charge carrier density provided by the Ag NPs and the vastly improved mobility afforded by the formation of continuous conductive pathways, a finding that aligns with established models for electron transport in nanocomposites [62].

**Figure 9:**
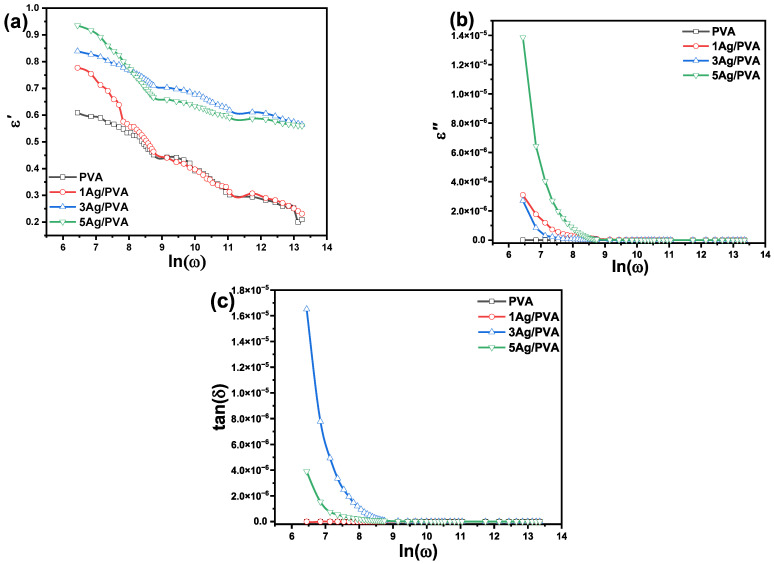
Frequency dependence of the (**a**) real ($\varepsilon^{\prime }$) and (**b**) imaginary (ε″) parts of the complex permittivity for pure PVA and Ag/PVA nanocomposites. (**c**) loss tangent (tan δ) for pure PVA and Ag/PVA nanocomposites.

The dielectric properties of the Ag/PVA nanocomposites were analyzed as a function of frequency (f) by examining the complex permittivity, comprising the real ($\varepsilon^{\prime }$) and imaginary ($\varepsilon^{\prime \prime }$) components, as depicted in Figure 9. The real part ($\varepsilon^{\prime }$), representing the dielectric constant, demonstrates a marked decline with rising frequency before plateauing in the high-frequency regime. Quantitatively, the ε′ value for pure PVA drops from 0.6 at 100 Hz to approximately 0.21 at 90 kHz. This trend is also evident in the Ag/PVA nanocomposites, with the 5 wt% Ag/PVA sample decreasing from 0.93 at 100 Hz to about 0.56 at the same high-frequency (at 90 kHz). The frequency-dependent behavior aligns with established principles for polymer composites [22], where the high low-frequency permittivity is governed by interfacial polarization and the successful alignment of molecular dipoles with the applied electric field. As the frequency increases, these polarization mechanisms become progressively unable to follow the rapid field reversals, leading to the observed saturation of the dielectric constant. This is a universal phenomenon in dielectric materials. At low frequencies, the dipolar groups within the PVA chains and the mobile charge carriers have sufficient time to align themselves with the alternating electric field, leading to a maximal response to a high response and high $\varepsilon^{\prime }$ values. As the frequency rises, the field alternates too rapidly for these dipoles to follow, causing their contribution to polarization to diminish and the dielectric constant to drop. Stabilization at high frequencies indicates a point where only atomic and electronic polarizations, which are instantaneous, can respond. The overall enhancement in ε′ values for the nanocomposites, particularly at low frequencies, can be linked to the introduction of additional defect sites and interfacial regions by the Ag NPs, which act as new charge trapping centers and increase space-charge polarization, consistent with prior studies [63]. The imaginary part ($\varepsilon^{\prime \prime }$), or dielectric loss, shows high values in the low-frequency regime. The observed high value of $\varepsilon^{\prime \prime }$ is primarily attributed to the long-range migration of mobile charge carriers (ions and electrons) and energy dissipation associated with interfacial polarization at the boundaries between the conductive Ag NPs and the insulating PVA matrix. At high frequencies, this ion diffusion is suppressed, leading to a reduction in $\varepsilon^{\prime \prime }$. Further analysis using the dipolar relaxation model, often represented by the loss tangent ($tan\;(\delta )\;=\;\varepsilon^{\prime \prime }/\varepsilon^{\prime }$), provides information on molecular mobility. The observation that tan δ decreases with both increasing frequency and Ag concentration indicates a shift in relaxation dynamics [64]. The assigned relaxation times suggest that the presence of Ag NPs influences the polymer’s chain dynamics. The dipolar entities in the PVA side chains align with the field at frequencies determined by a balance between their inherent elastic restoring forces and the frictional damping from the surrounding polymer environment [65]. The Ag NPs appear to restrict this molecular motion, potentially through physical crosslinking, thereby altering the characteristic relaxation frequencies and contributing to the observed dielectric profile.

**Figure 10:**
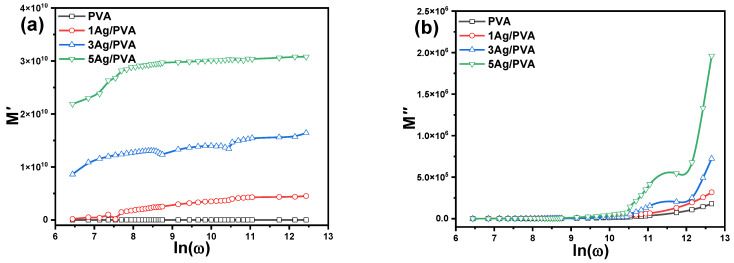
Frequency dependence of the (**a**) real ($M^{\prime }$) and (**b**) imaginary (M″) parts of the complex electric modulus for pure PVA and Ag/PVA nanocomposites.

The analysis of the complex electric modulus ($M^{*}=\;M^{\prime }+iM\prime \prime$) provides a powerful tool to understand the dielectric relaxation dynamics and bulk electrical properties of the Ag/PVA nanocomposites, while effectively suppressing the confounding effects of electrode polarization. The frequency-dependent behavior of $M^{\prime }$ and M″ for Ag/PVA composites with varying Ag concentrations is presented in Figure 10.

A key observation is the relatively low value of the real part, $M^{\prime }$, which remains around 30 across the frequency spectrum. This consistently low magnitude confirms that the contribution of electrode polarization to the overall dielectric response is negligible, allowing for a clearer interpretation of the intrinsic material properties [59,60,62]. This validates that the observed phenomena are indeed related to the bulk relaxation processes within the nanocomposite. The behavior of the imaginary part, M″, offers critical insight into the charge transport dynamics. A distinct, asymmetric peak is observed in the M″ spectrum, located within the dispersion region of $M^{\prime }$. This peak is associated with the conductivity relaxation frequency, a characteristic timescale for the decay of the electric field within the material. As the Ag NPs concentration increases up to 5 wt%, this M″ peak systematically shifts towards higher frequencies. This shift signifies a decrease in the conductivity relaxation time, meaning that the dipolar entities and charge carriers within the nanocomposite can realign themselves more rapidly in response to the alternating electric field. This trend indicates a shortening of the dipolar relaxation time and an overall improvement in the dynamic electrical response with higher Ag loading. However, a notable deviation occurs at the 5 wt% Ag concentration, where a decline in overall conductivity is observed despite the faster relaxation. This counterintuitive result is likely attributed to the incipient agglomeration of Ag NPs at this higher Ag loading. Agglomeration can disrupt the formation of continuous conductive pathways, creating microstructural defects that hinder long-range charge transport, even while the local, short-range polarization dynamics are accelerated. Thus, the electric modulus analysis successfully reveals a complex interplay between enhanced local dipole mobility and the macroscopic percolation network, which can be compromised by nanoparticle aggregation at critical filler concentrations [66].

The calculation of the electric modulus is primarily used to minimize the contribution of electrode polarization. Analysis of M″ reveals a characteristic asymmetric peak, from which the dipole relaxation time ($\tau_{p}$) can be determined using the relation: $\tau_{p}\;=\;\frac{1}{\omega_{p}}$, where $\omega_{p}$ is the angular frequency at the peak maximum. The value of τ was determined to be 22.78 µs for pure PVA and 20.51 for µs for 5 wt% Ag/PVA nanocomposite. For the other Ag/PVA nanocomposite samples, however, the $M^{\prime \prime }$ peak was not observed within the measured frequency range. The consistently low values of $M^{\prime }$ confirm the suppression of electrode effects. Notably, the M″ peak shifts to higher frequencies with increasing Ag ratio, indicating a decrease in relaxation time, which agrees with other studies [22].

**Figure 11:**
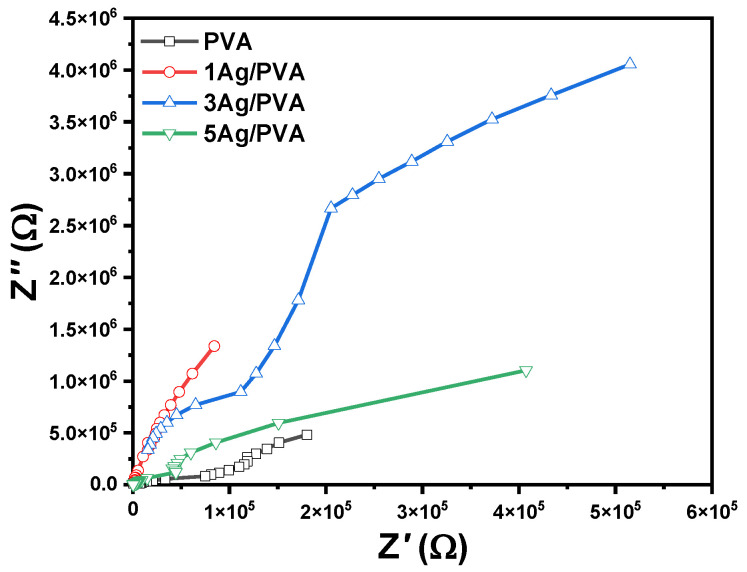
Nyquist plots ($-Z^{\prime \prime }$ versus $\;Z^{\prime }$) for pristine PVA and Ag/PVA nanocomposites with varying Ag concentrations.

The electrical impedance characteristics of the Ag/PVA nanocomposites were further elucidated through Nyquist plots (−Z″ versus $Z^{\prime }$), which provide a powerful means to visualize the frequency-dependent conduction mechanisms and interfacial phenomena. The plots, presented in Figure 11, reveal a clear evolution in the electrical behavior with increasing Ag NPs content. The Nyquist spectrum for pure PVA is characterized by a very high overall magnitude and an incomplete semicircular arc, which is a classic signature of a material with high electrical resistance and predominantly insulating behavior. This indicates that charge carriers in pure PVA are largely immobilized, resulting in minimal conductive response. A significant transformation is observed upon the incorporation of Ag NPs. The diameter of the semicircular arc in the Nyquist plot systematically decreases with higher Ag concentrations. The reduction in the arc radius corresponds directly to a substantial decrease in the bulk resistance of the nanocomposite films. The detected trend is complemented by a noticeable shift of the high-frequency dispersion region toward higher frequencies. Also, this shift signifies a decrease in the conductivity relaxation time, meaning that charge carriers can respond more rapidly to the changing electric field. The well-fitted, depressed semicircles for the nanocomposite samples suggest a conduction mechanism that can be accurately modeled using a parallel resistor-capacitor equivalent circuit, representing the bulk material properties. The consistent decrease in both the real ($Z^{\prime }$) and imaginary (Z″) components of impedance with increasing frequency further confirms the enhanced mobility of charge carriers. At higher frequencies, the capacitive elements in the composite offer less impedance, allowing for easier transport of charge and leading to the observed improvement in AC conductivity. Collectively, the impedance analysis provides compelling evidence that the dispersed Ag NPs create conductive pathways, facilitating charge transport and significantly lowering the relaxation time, thereby enhancing the overall electrical conductivity of the PVA matrix [67].

The stability of the nanocomposites against ambient oxidation is a critical consideration. The in situ synthesis, where PVA acts as a stabilizer, and the embedded nature of the Ag NPs within the dense polymer matrix are believed to significantly mitigate direct exposure to atmospheric oxygen and moisture. This is supported by the consistent and high performance of the composites (e.g., mechanical strength, electrical conductivity) measured over the experimental timeframe, indicating minimal degradation under standard laboratory conditions. Future work will include dedicated long-term aging studies to quantify stability over extended periods.

### 3.7. Surface Hydrophilic and Antibacterial Properties

**Figure 12:**
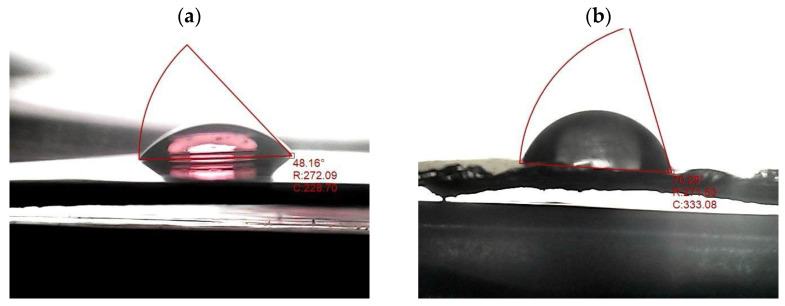

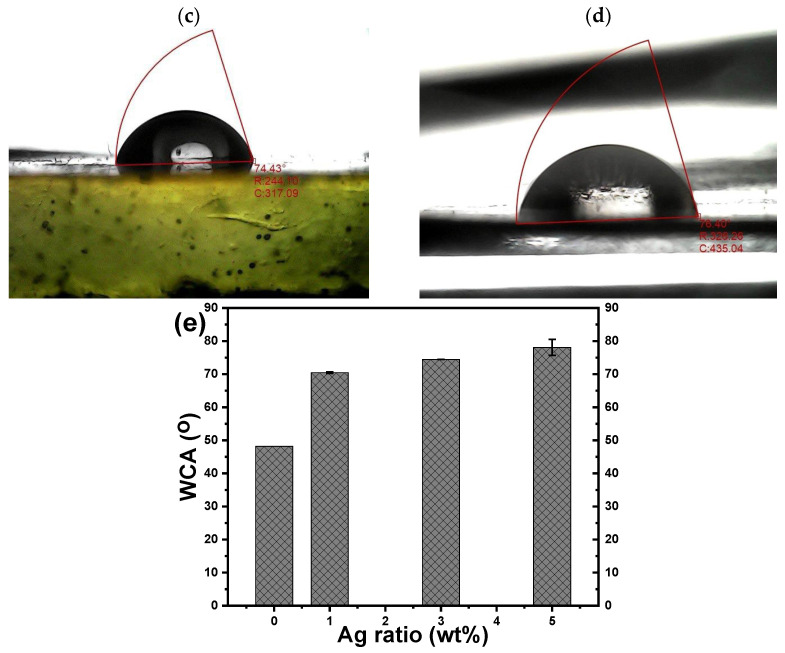
Optical images of water droplets on (**a**) pure PVA and Ag/PVA composites with Ag ratios of (**b**) 1 wt%, (**c**) 3 wt%, and (**d**) 5 wt%. (**e**) The corresponding quantitative WCA values as a function of Ag NPs concentration.

The surface wettability of a material, a critical property in fields ranging from materials engineering to chemistry, is fundamentally governed by the interplay between surface energy and topography. PVA is a hydrophilic polar polymer with abundant hydroxyl (–OH) groups along its backbone. These groups readily form hydrogen bonds with water molecules, resulting in a strong affinity for water. Figure 12a,d show optical images of water droplets on pure PVA and Ag/PVA composites. The corresponding water contact angle (WCA) values are plotted as a function of Ag NPs concentration in Figure 12e. This inherent hydrophilicity was quantitatively confirmed by WCA measurement of approximately 48.16° for the pure PVA film. This low contact angle is consistent with PVA’s known water-soluble and biodegradable nature, which makes it suitable for various solution-based processing techniques [68]. The incorporation of Ag NPs, however, induces a significant and systematic modification of the PVA surface properties. Wettability analysis of the Ag/PVA nanocomposite films with Ag loadings of 1, 3, and 5 wt% revealed a clear trend: the water contact angle increased progressively with higher Ag content from 48.16° to 78° ± 2.4° for 5 wt% Ag/PVA nanocomposite. The inverse relationship between Ag concentration and surface wettability indicates a transition towards a more hydrophobic surface character. This phenomenon can be attributed to two main factors. First, the hydrophobic nature of the metallic Ag NPs themselves reduces the overall surface energy of the Ag/PVA nanocomposite. As the Ag content increases, these Ag NPs occupy more of the film’s surface, presenting a barrier that disrupts the hydrogen-bonding network of the PVA matrix. Second, the embedding of Ag NPs within the PVA polymer can alter the surface topography and roughness at the micro- and nanoscale. The presence of Ag NPs can create a composite interface that reduces the effective contact area between the water droplet and the hydrophilic PVA, thereby facilitating a higher contact angle. The detected hydrophobic modification, achieved through simple compositional tuning, expands the potential application scope of PVA-based materials, particularly for flexible electronic devices where controlled moisture resistance may be required.

**Figure 13:**
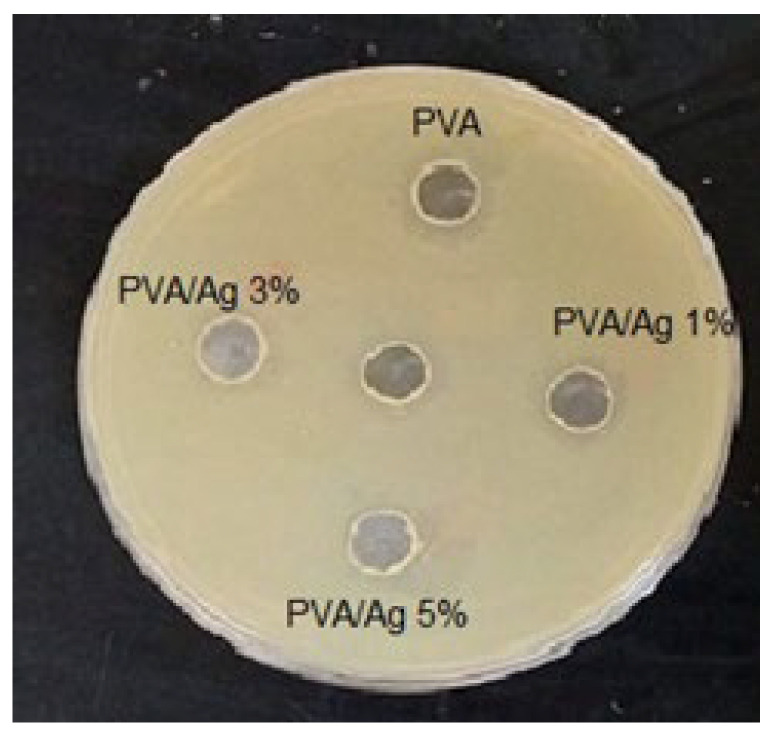
Results of the disc diffusion antibacterial assay against *E. Coli* for pure PVA and Ag/PVA nanocomposites showing the corresponding inhibition zones. The central zone in the panel is the position of the respective film sample.

The antibacterial activity of the synthesized PVA and Ag/PVA composite films was quantitatively assessed against the Gram-negative bacterium Escherichia coli (*E. coli*) using the disc diffusion method, with the results visualized in Figure 13. The assay provided a clear distinction between the pure PVA and the Ag NPs-loaded composites. As anticipated, the pure PVA film showed no inhibitory effect, evident from the absence of a clearance zone around the sample disc. This confirms the inherent biocompatibility and lack of intrinsic bactericidal properties in the PVA polymer matrix. In stark contrast, all Ag/PVA nanocomposites demonstrated significant and dose-dependent antibacterial activity. A clear inhibition zone of 1.1 cm was observed for the composite containing 1 wt% Ag. The antibacterial effect intensified with increasing silver content, with the inhibition zone expanding to 1.8 cm for the 3 wt% Ag composite and reaching 2.0 cm for the sample with the highest loading of 5 wt% Ag. This progressive enhancement in bactericidal performance is a hallmark of Ag NPs activity. The mechanism is primarily attributed to the sustained release of Ag ions (Ag^+^) from the nanoparticle surface, which readily penetrates and disrupts key bacterial cellular functions. These Ag ions can bind to thiol groups in vital enzymes, deactivating them, and also generate reactive oxygen species that cause oxidative damage to cell membranes and internal structures. The direct correlation between the larger inhibition zones and higher Ag loadings indicates a greater bioavailability of these biocidal Ag ions. These compelling results confirm that the incorporation of Ag NPs successfully endows the PVA hydrogel with strong and tailorable antibacterial properties, underscoring its significant potential for use in wound dressings, antimicrobial coatings, and other biomedical applications where preventing microbial growth is critical.

In contrast to our study, which demonstrated a clear, dose-dependent antibacterial effect against *E. coli* with Ag concentrations as low as 1 wt%, other synthesis methods show different profiles. For instance, Ag/PVA nanofibers made by electrospinning were more effective against S. aureus than *E. coli* [69], while plasma-treated Ag/PVA only exhibited significant activity after a critical 20 min processing time [56]. The above analysis highlights that our method achieves potent and consistent antibacterial activity at lower Ag loadings, without requiring a prolonged activation step.

The observed inhibition zones indicate the diffusion of a bactericidal component from the composite films. While this study does not isolate the specific mechanism, the diffusion-mediated activity is consistent with the well-established release of Ag^+^ from nanocomposites, which is a primary pathway for the antimicrobial action of Ag NPs [70]. A direct distinction between this ion-release mechanism and a contact-killing effect would require further analysis, such as measuring ion release kinetics or using direct-contact assays. Furthermore, the antibacterial activity reported here is specific to the Gram-negative model organism *E. coli*. Evaluating performance against Gram-positive bacteria (e.g., Staphylococcus aureus) would be necessary to fully define the material’s antimicrobial spectrum and is an important direction for future research.

## 4. Conclusions

In this study, multifunctional Ag/PVA nanocomposite films were successfully synthesized via a solution casting technique, with Ag NP concentrations systematically varied from 1 wt% to 5 wt%. A comprehensive characterization confirmed that the incorporation of Ag NPs profoundly and beneficially altered the properties of the PVA matrix. Structurally, FTIR analysis confirmed the successful formation of the Ag/PVA composites, indicating an interaction between the Ag NPs and the PVA polymer’s hydroxyl groups. The studied materials exhibited a pronounced red shift in the surface plasmon resonance band and a significant reduction in the optical band gap of PVA from 3.78 eV to 3.37 eV, demonstrating enhanced light–matter interaction and tunable electronic properties. The surface character shifted from hydrophilic to more hydrophobic, as evidenced by the increase in water contact angle from 48.16° to over 79°, which is advantageous for moisture resistance in applications. The Ag/PVA nanocomposites displayed substantial reinforcement, with marked increases in tensile strength, Young’s modulus, and surface hardness, attributed to the Ag NPs acting as crosslinking points within the polymer network. The electrical performance was dramatically enhanced, with AC conductivity increasing by several orders of magnitude and dielectric properties confirming the formation of conductive pathways. The thermal conductivity saw a significant improvement, rising from 0.27 W/m·K for pure PVA to 0.92 W/m·K for the 5 wt% Ag/PVA composites. The composites exhibited strong, concentration-dependent antibacterial activity against *E. coli*, transforming the inert PVA into an effective bactericidal material. Collectively, these results provide a unified and quantitative framework that fundamentally differentiates this work: it establishes clear, causal structure–property relationships by demonstrating how a single variable, Ag NPs concentration, systematically modulates a comprehensive suite of functionalities within a simplified binary system. This insight moves beyond reporting property enhancements to offering a foundational guide for the rational design of Ag/PVA composites. Therefore, the synergistic enhancement of optical, mechanical, electrical, thermal, and antibacterial properties underscores the high potential of these Ag/PVA nanocomposites for advanced applications in flexible electronics, antimicrobial coatings, and smart packaging.

## Figures and Tables

**Table 1:** FTIR band assignments for pure PVA and 5 wt% Ag/PVA nanocomposite.

Wavenumber (cm^−1^)	Assignment
~3300	O–H Stretching
~2930 & 2940	CH_2_ asymmetric stretching
~1710 & 1652	C=O stretching
~1429 & 1326	CH_2_ bending and C–H wagging
~1293 & 1105	C–O stretching
~1081 & 1066	Alkoxy C–O stretching
~845–370	Coordination between Ag and PVA matrix

**Table 2:** Summary of the measured properties of Ag/PVA nanocomposites: optical energy band gaps (E_g1_, E_g2_), thermal conductivity (K_t_), mechanical properties (tensile stress, strain, Young’s modulus, hardness Shore A), and AC electrical conductivity ($\sigma_{ac}$).

Sample	E_g1_ (eV)	E_g2_ (eV)	K_t_ (W/m^2^ K)	Stress (N/mm^2^)	Strain (%)	Y_M_ (MPa)	Hardness Shore A	$\sigma_{ac}$ (S/cm)
PVA	3.78	−	0.27	4.1	3.2	1.2 ± 0.01	52 ± 0.2	5.8 × 10^−9^
1 wt% Ag/PVA	3.66	2.41	0.36	5.58	3.3	1.5 ± 0.01	55 ± 0.2	1.2 × 10^−7^
3 wt% Ag/PVA	3.38	2.37	0.81	19.92	3.1	1.7 ± 0.01	60 ± 0.3	1.3 × 10^−6^
5 wt% Ag/PVA	3.37	2.37	0.92	30.5	3.06	2.4 ± 0.02	68 ± 0.1	1.01 × 10^−4^

## Data Availability

The original contributions presented in this study are included in the article. Further inquiries can be directed to the corresponding authors.
